# Coral-Derived Compound WA-25 Inhibits Angiogenesis by Attenuating the VEGF/VEGFR2 Signaling Pathway

**DOI:** 10.3390/md13020861

**Published:** 2015-02-06

**Authors:** Shih-Wei Lin, Shih-Chung Huang, Hsiao-Mei Kuo, Chiu-Hua Chen, Yi-Ling Ma, Tian-Huei Chu, Youn-Shen Bee, E-Ming Wang, Chang-Yi Wu, Ping-Jyun Sung, Zhi-Hong Wen, Deng-Chyang Wu, Jyh-Horng Sheu, Ming-Hong Tai

**Affiliations:** 1 Doctoral Degree Program in Marine Biotechnology, National Sun Yat-Sen University and Academia Sinica, Kaohsiung 804, Taiwan; E-Mails: shiwey@yahoo.com.tw (S.-W.L.); wzh@mail.nsysu.edu.tw (Z.-H.W.); 2 Institute of Biomedical Sciences, National Sun Yat-sen University, Kaohsiung 804, Taiwan; E-Mails: sghung@hotmail.com (S.-C.H.); skbboyz0817@gmail.com (T.-H.C.); 3 Department of Internal Medicine, Kaohsiung Armed Forces General Hospital, Kaohsiung 802, Taiwan; 4 Mitochondrial Research Unit, Kaohsiung Chang Gung Memorial Hospital and Chang Gung University College of Medicine, Kaohsiung 833, Taiwan; E-Mail: hsiaomeikuo@gmail.com; 5 Division of Nephrology Department of Internal Medicine, Kaohsiung Chang Gung Memorial Hospital and Chang Gung University College of Medicine, Kaohsiung 833, Taiwan; E-Mail: becky0917@yahoo.com.tw; 6 Department of Biological Sciences, National Sun Yat-Sen University, Kaohsiung, Taiwan; E-Mails: ylma@vghks.gov.tw (Y.-L.M.); emwang@vghks.gov.tw (E.-M.W.); cywu@mail.nsysu.edu.tw (C.-Y.W.); 7 Division of Nephrology, Kaohsiung Veterans General Hospital, Kaohsiung 813, Taiwan; 8 Department of Ophthalmology, Kaohsiung Veterans General Hospital, Kaohsiung 804, Taiwan; E-Mail: ysbee@vghks.gov.tw; 9 National Museum of Marine Biology and Aquarium, Pingtung 944, Taiwan; E-Mail: pjsung@nmmba.gov.tw; 10 Department of Marine Biotechnology and Resources National Sun Yat-Sen University, Kaohsiung 804, Taiwan; 11 Center for Stem Cell Research, Kaohsiung Medical University, Kaohsiung 807, Taiwan; E-Mail: dechwu@yahoo.com; 12 Division of Gastroenterology, Department of Internal Medicine, Kaohsiung Medical University Hospital, Kaohsiung 807, Taiwan; 13 Department of Medicine, Faculty of Medicine, College of Medicine, Kaohsiung Medical University, Kaohsiung 807, Taiwan; 14 Department of Medical Research, China Medical University Hospital, China Medical University, Taichung 404, Taiwan

**Keywords:** marine drugs, human umbilical vein endothelial cells (HUVECs), vascular endothelial growth factor (VEGF), VEGF receptor 2 (VEGFR2)

## Abstract

**Background**: WA-25 (dihydroaustrasulfone alcohol, a synthetic derivative of marine compound WE-2) suppresses atherosclerosis in rats by reducing neointima formation. Because angiogenesis plays a critical role in the pathogenesis of atherosclerosis, the present study investigated the angiogenic function and mechanism of WA-25. **Methods**: The angiogenic effect of WA-25 was evaluated using a rat aortic ring assay and transgenic zebrafish models were established using transgenic *Tg*(*fli-1:EGFP*)*^y1^* and *Tg(kdrl:mCherry^ci5^-fli1a:negfp^y7^)* zebrafish embryos. In addition, the effect of WA-25 on distinct angiogenic processes, including matrix metalloproteinase (MMP) expression, endothelial cell proliferation and migration, as well as tube formation, was studied using human umbilical vein endothelial cells (HUVECs). The effect of WA-25 on the endothelial vascular endothelial growth factor (VEGF) signaling pathway was elucidated using qRT-PCR, immunoblot analysis, immunofluorescence and flow cytometric analyses. **Results**: The application of WA-25 perturbed the development of intersegmental vessels in transgenic zebrafish. Moreover, WA-25 potently suppressed microvessel sprouting in organotypic rat aortic rings. Among cultured endothelial cells, WA-25 significantly and dose-dependently inhibited MMP-2/MMP-9 expression, proliferation, migration and tube formation in HUVECs. Mechanistic studies revealed that WA-25 significantly reduced the VEGF release by reducing VEGF expression at the mRNA and protein levels. In addition, WA-25 reduced surface VEGF receptor 2 (VEGFR2/Flk-1) expression by repressing the VEGFR2 mRNA level. Finally, an exogenous VEGF supply partially rescued the WA-25-induced angiogenesis blockage *in vitro* and *in vivo*. **Conclusions**: WA-25 is a potent angiogenesis inhibitor that acts through the down-regulation of VEGF and VEGFR2 in endothelial cells. **General** **Significance**: WA-25 may constitute a novel anti-angiogenic drug that acts by targeting endothelial VEGF/VEGFR2 signaling.

## 1. Introduction

In the history of drug discovery, marine-derived extracts or compounds have served as unique pharmaceutical sources and gained increasing attention in recent years for the treatment of intractable diseases [1]. For example, ziconotide from cone snails has been approved for the treatment of intractable pain in patients with spinal injuries [2,3]. Many countries are conducting large-scale screening of marine compounds, particularly for the development of anticancer [4,5], antimalarial [6], antiviral [7] and anti-inflammatory drugs [8,9].

Angiogenesis, the formation of new capillaries from the existing vasculature, is a key event in physiological (wound healing and developmental progress) and pathological (tumor growth and metastasis) conditions [10,11]. Angiogenesis inhibition constitutes a novel therapeutic strategy for several human diseases, including cancer, inflammation, cardiac hypertrophy [10], peripheral arterial disease [11], and ischemic heart diseases [12]. Angiogenesis involves a multiple cellular processes including endothelial cell proliferation, migration, and morphological differentiation and is closely regulated by growth factors and intracellular signaling pathways [13,14]. Vascular endothelial growth factor (VEGF) is viewed as one of the most critical angiogenic mediators [15,16,17]. VEGF secreted from tumor and endothelial cells plays critical roles in tumor progression, particularly in tumor angiogenesis and metastasis [18,19,20]. Moreover, VEGF and VEGF receptors, particularly VEGF receptor 2 (VEGFR2/Flk-1), are considered to constitute the key signaling system regulating endothelial cell proliferation and migration [14,21,22]. Therefore, the suppression of VEGF signaling pathway is considered a potential strategy for tumor angiogenesis inhibition. 

WA-25 (dihydroaustrasulfone alcohol) is a synthetic intermediate in the total synthesis of the marine compound WE-2 (austrasulfone), an anti-inflammatory compound isolated from the soft coral *Cladiella australis* [23]. The anti-inflammatory function of WA-25 may be attributed to its capability of inhibiting the expression of inducible nitric oxide synthetase (iNOS) and cyclooxygenase-2 (COX-2) in endotoxin-stimulated macrophage cells [23]. Moreover, WA-25 administration potently reduces the balloon injury-induced neointima formation in rat model of atherosclerosis, further supporting its anti-inflammatory role. However, the mechanism underlying the anti-atherosclerotic function of WA-25 remains unclear. Because angiogenesis occurs in neointima formation during atherosclerosis, the present study first investigated the function of WA-25 in angiogenesis by using animal models. Subsequently, the anti-angiogenic function and mechanism of WA-25 were delineated using cultured endothelial cells.

## 2. Results

### 2.1. WA-25 Perturbs Vessel Development in Zebrafish and Rat Aortic Rings

**Figure 1:**
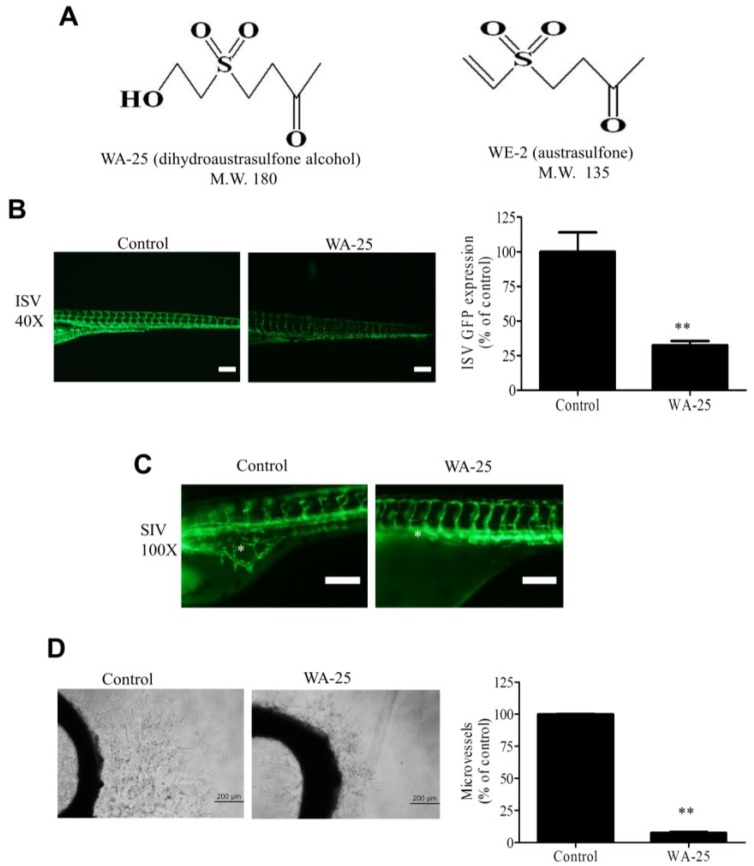
Angiogenesis inhibition by WA-25 *in vivo* and *ex vivo* (**A**) Chemical structures of WA-25 (dihydroaustrasulfone alcohol) and WE-2 (austrasulfone); (**B**) Effect of WA-25 on intersegmental vessels (ISVs) development in transgenic *Tg*(*fli-1:EGFP*)^y1^ zebrafish embryos. (Left panel) Representative photographs of ISV in control and WA-25 (50 μM)-treated zebrafish at 48 h poat-fertilization (hpf). Magnification 40×; scale bar, 50 μm. (Right panel) Quantification analysis of enhanced green fluorescent protein (EGFP) intensities in the ISVs in WA-25 (50 μM)-treated embryos. Data are represented as mean ± SEM (*n* = 12); (**C**) Effect of WA-25 (50 μM) on SIV development in transgenic *Tg*(*fli-1:EGFP*)^y1^ zebrafish embryos. Photographs of control and WA-25-treated zebrafish were taken at 72 hpf. Magnification, 100×; scale bar, 100 μm. Asterisks indicate arcades in the vesicle-like structure (**D**) Effect of WA-25 on microvessel sprouting in aortic rings. Rat aortic rings were placed in Matrigel and treated with WA-25 (20 μM) for 7 days. Scale bar, 2 mm. Data are represented as mean ± SEM (*n* = 12). ** *p* < 0.01.

To evaluate the influence of WA-25 on angiogenesis, we employed the transgenic *Tg*(*fli-1:EGFP*)^y1^ zebrafish model, which expresses the enhanced green fluorescent protein (EGFP) in blood vessels. We found that WA-25 supply prominently reduced EGFP expression in the intersegmental vessels (ISVs) of zebrafish embryos (Figure 1B). The quantification analysis revealed that WA-25 treatment significantly reduced the fluorescence intensities by approximately 70% compared with the control. In addition, a continuous WA-25 exposure further disrupted the formation of neovascularized network in the subintestinal vessel plexus (SIV) of zebrafish (Figure 1C). Nevertheless, WA-25 treatment was highly tolerated because all zebrafish embryos remained viable for at least seven days. Subsequently, we evaluated the antiangiogenic effect of WA-25 by using rat organotypic aortic rings. We observed that the application of WA-25 significantly perturbed microvessel sprouting in rat aortic rings by more than 80% of that in control (Figure 1D). Overall, these findings indicate that WA-25 inhibits vessel development in various animal models.

### 2.2. WA-25 Inhibits Matrix Metalloproteinase Secretion, Proliferation, Migration and Tube Formation of Cultured Endothelial Cells

**Figure 2:**
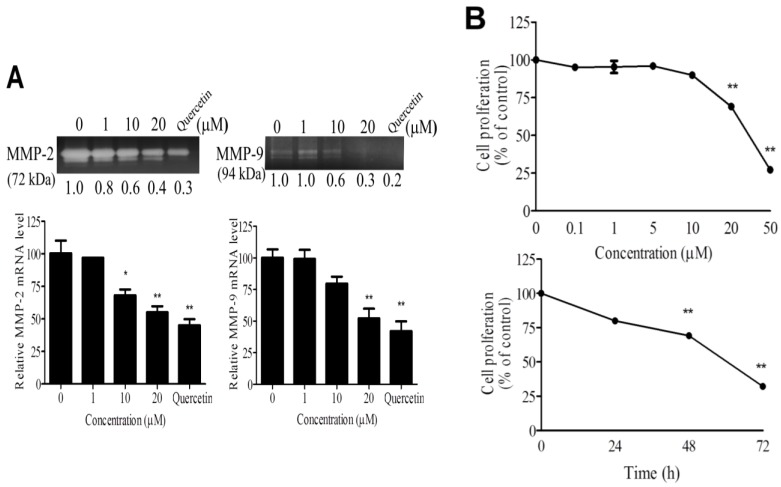

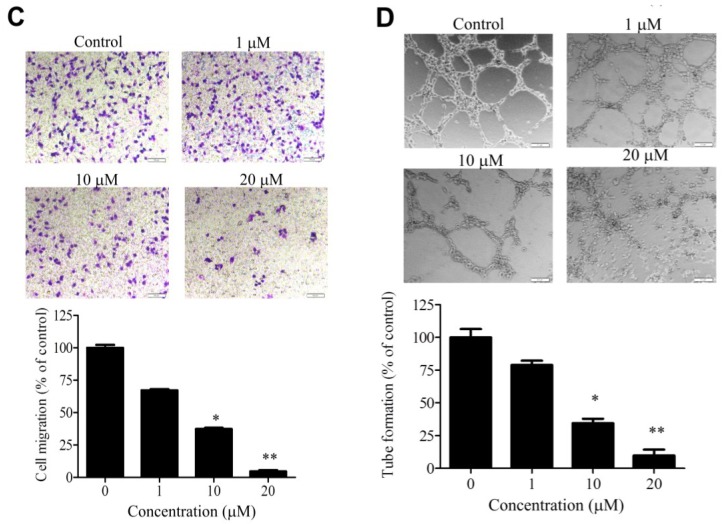
Effects of WA-25 on angiogenic processes of endothelial cells (**A**) Effect of WA-25 on matrix metalloproteinase (MMP) secretion. After incubation with WA-25 (1–20 μM) or Quercetin (50 μM) for 48 h, the conditioned media of human umbilical vein endothelial cells (HUVECs) were analyzed for MMP-2 and MMP-9 activities by using gelatin zymography and for the mRNA level by using qRT-PCR, WA-25 reduced the MMP-2 and MMP-9 mRNA levels in HUVECs. Data are represented as the average fold over control from three independent experiments; (**B**) Effect of WA-25 on endothelial cell proliferation. HUVECs were treated with various doses of WA-25 (0.1–50 μM) in 10% serum medium for (0–72 h). HUVEC proliferation was measured using 3-[4,5-dimethylthiazol-2-yl]-2,5-diphenyl-tetrazolium bromide (MTT) assay and expressed as mean ± SD percentages of control in triplicate; (**C**) Effect of WA-25 on endothelial cell migration. After treatment with WA-25 (1–20 μM), the effects of WA-25 on HUVEC migration were examined using the Boyden chamber transwell assay; (**D**) Effect of WA-25 on tube formation in endothelial cells. After plating on Matrigel-coated wells, the tube formation of HUVECs treated with WA-25 (1–20 μM) was recorded after 8 h. Data are represented as mean ± SD percentages of the control in triplicate. Asterisks indicate statistical significance *versus* control (* *p* < 0.05 and ** *p* < 0.01).

To delineate the antiangiogenic effect of WA-25 on distinct angiogenic processes, we investigated the effects of WA-25 on matrix metalloproteinase (MMP) secretion, proliferation, migration, and tube formation in cultured endothelial cells. By using gelatin zymography analysis and Quercetin as a positive control of MMP inhibitors [24,25], we found that WA-25 treatment prominently attenuated MMP-2 and MMP-9 activities in cultured endothelial cells (Figure 2A). Besides, the quantitative reverse transcription-polymerase chain reaction (qRT-PCR) analysis indicated that WA-25 inhibited the MMP-2 and MMP-9 expression at transcriptional levels. Furthermore, WA-25 treatment significantly inhibited endothelial cell proliferation in a dose-dependent manner, with a half-maximal inhibitory concentration (IC_50_) of 27.8 μM (Figure 2B). In a Boyden chamber migration assay, WA-25 treatment perturbed endothelial cell migration with an IC_50_ of 8.9 μM (Figure 2C). Finally, WA-25 dose-dependently disrupted the formation of a tube-like structure in HUVECs in the Matrigel with an IC_50_ of 5.83 μM (Figure 2D). Overall, these results indicate that WA-25 suppresses the MMP release, proliferation, migration and tube formation in endothelial cells.

### 2.3. WA-25 Reduces the Release and Alleviates the Bioavailability of Vascular Endothelial Growth Factor-A in Endothelial Cells

**Figure 3:**
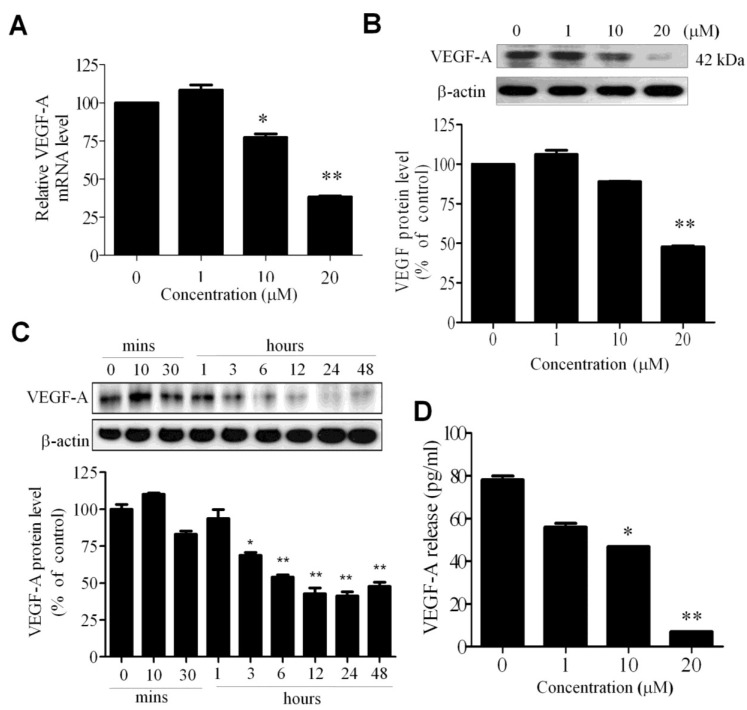
Effect of WA-25 on VEGF expression in endothelial cells. After treatment with WA-25 (1–20 μM) for 48 h, HUVECs were analyzed for VEGF-A mRNA and protein levels. (**A**) Quantitative qRT-PCR analysis of the VEGF mRNA level; (**B**) Immunoblot analysis of the dose-dependent effect of WA-25 on the VEGF-A protein level; (**C**) Immunoblot analysis of the time-dependent effect of WA-25 on the VEGF protein level; (**D**) ELISA of VEGF secretion in HUVECs after treatment with WA-25 for 48 h. Data are represented as mean ± SD in triplicates. * *p* < 0.05; ** *p* < 0.01.

Because VEGF-A signaling plays a pivotal role in angiogenesis, we investigated whether WA-25 exerted an antiangiogenic effect through the modulation of VEGF-A expression in endothelial cells. The qRT-PCR analysis revealed that WA-25 reduced the VEGF-A mRNA level in HUVECs (Figure 3A). A Western blot analysis further revealed that WA-25 reduced the VEGF-A protein level in a dose—(Figure 3B) and time-dependent manner (within 3 h of the treatment; Figure 3C). Finally, an enzyme-linked immunosorbent assay (ELISA) revealed that WA-25 significantly reduced VEGF-A secretion in HUVECs (Figure 3D). Overall, these results indicate that WA-25 disturbs the VEGF-A homeostasis in endothelial cells at the transcriptional level.

### 2.4. WA-25 Reduces Vascular Endothelial Growth Factor Receptor 2 Expression in Endothelial Cells

**Figure 4:**
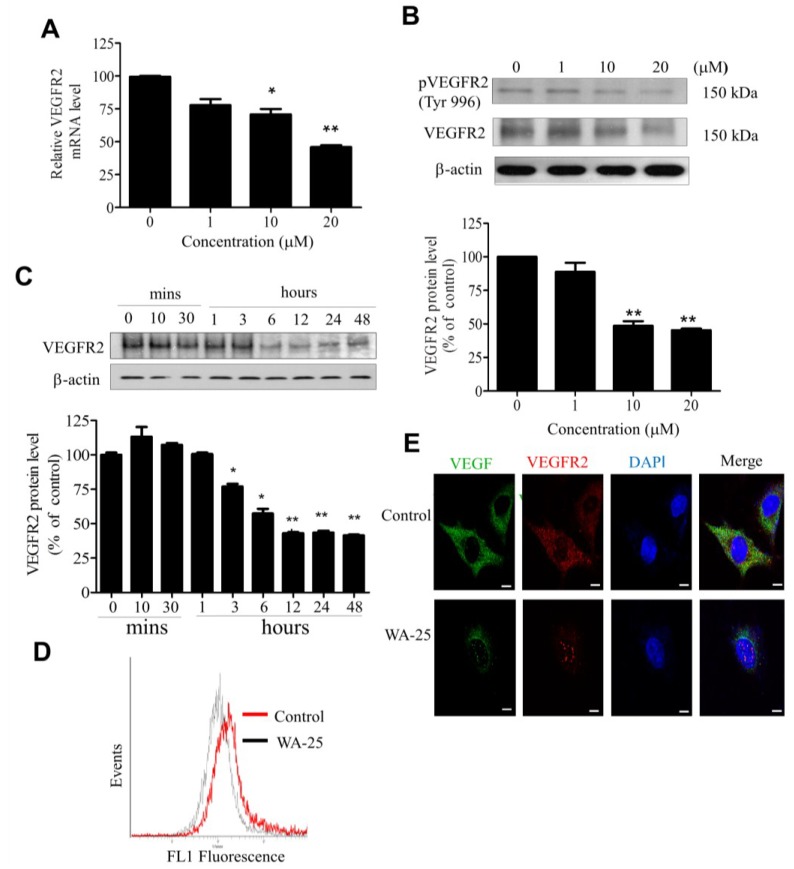
Effect of WA-25 on VEGFR2 expression in HUVECs. HUVECs were treated with WA-25 (1–20 μM) for 48 h and separately subjected to VEGFR2 mRNA and protein expression assay. (**A**) VEGFR2 mRNA level was determined using quantitative qRT-PCR analysis. (**B**) Dose-dependent (48 h) and (**C**) time-dependent effect of WA-25 on VEGFR2 protein expression was measured using Western blot analysis (**D**) Flow cytometric analysis of surface VEGFR2 expression after WA-25 (20 μM) treatment for 48 h (**E**) VEGF and VEGFR2 protein expressions were analyzed using immunofluorescence. After WA-25 (20 μM) treatment for 48 h, the cell surface VEGFR2 expression in endothelial cells was analyzed using FACScan. Data are represented as mean ± SD in triplicates. * *p* < 0.05; ** *p* *<* 0.01, *versus* control groups.

Because VEGFR2 is the primary receptor mediating the VEGF angiogenic function in endothelial cells, we evaluated the influence of WA-25 on VEGFR2 expression by using qRT-PCR analysis, which showed that WA-25 significantly reduced the VEGFR2 mRNA level in endothelial cells (Figure 4A). Western blot analysis further demonstrated that WA-25 reduced VEGFR2 expression in endothelial cells in a dose- (Figure 4B) and time-dependent manner (within 12 h; Figure 4C). The flow cytometric analysis confirmed that WA-25 diminished the surface VEGFR2 expression in endothelial cells (Figure 4D), which was supported by the result of immunofluorescence analysis (Figure 4E). These results demonstrate that WA-25 elicited VEGFR2 down-regulation in endothelial cells.

### 2.5. Vascular Endothelial Growth Factor-A Supply Partially Rescues the WA-25-Induced Neovascularization Blockade in Vitro

**Figure 5:**
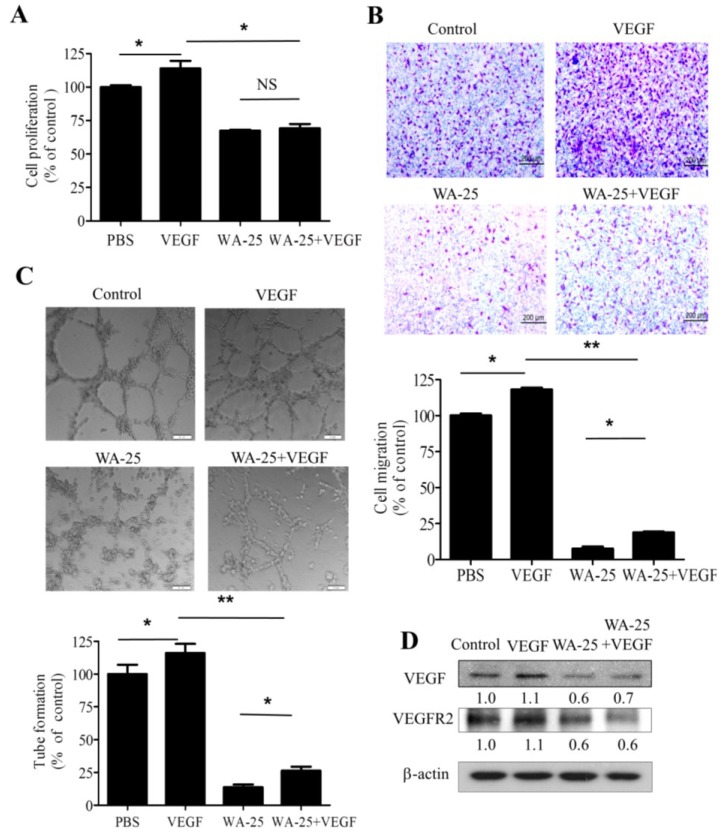
Effects of exogenous VEGF on WA-25-induced neovascularization blockade *in vitro*. After treatment with WA-25 (20 μM) with or without VEGF (10 ng/mL), the effects of WA-25 on VEGF-induced proliferation (**A**); migration (**B**); and tube formation (**C**) were determined in HUVECs; (**D**) Immunoblot analysis of VEGF and VEGFR2 expression in HUVECs after treatment with WA-25 (20 μM) with or without VEGF (10 ng/mL) for 24 h. Data are represented as mean ± SD of quadruplicate experiments. Asterisks indicate statistical significance *versus* control (* *p* < 0.05 and ** *p <* 0.01). NS, not significant.

Because WA-25 treatment reduced the cellular VEGF level in endothelial cells, we investigated whether exogenous VEGF could alleviate the antiangiogenic function of WA-25 in endothelial cells. We found that despite having no effect on proliferation (Figure 5A), exogenous VEGF partially alleviated the WA-25-induced inhibition of migration (Figure 5B) and tube formation (Figure 5C) in endothelial cells. Moreover, the immunoblot analysis revealed that VEGF supply partially restored the WA-25-induced VEGF down-regulation, but not VEGFR2. (Figure 5D). Thus, WA-25 is a potent antagonist of the VEGF signaling pathway.

### 2.6. Vascular Endothelial Growth Factor-A Partially Rescues the WA-25-Induced Neovascularization Blockade in Vivo

**Figure 6:**
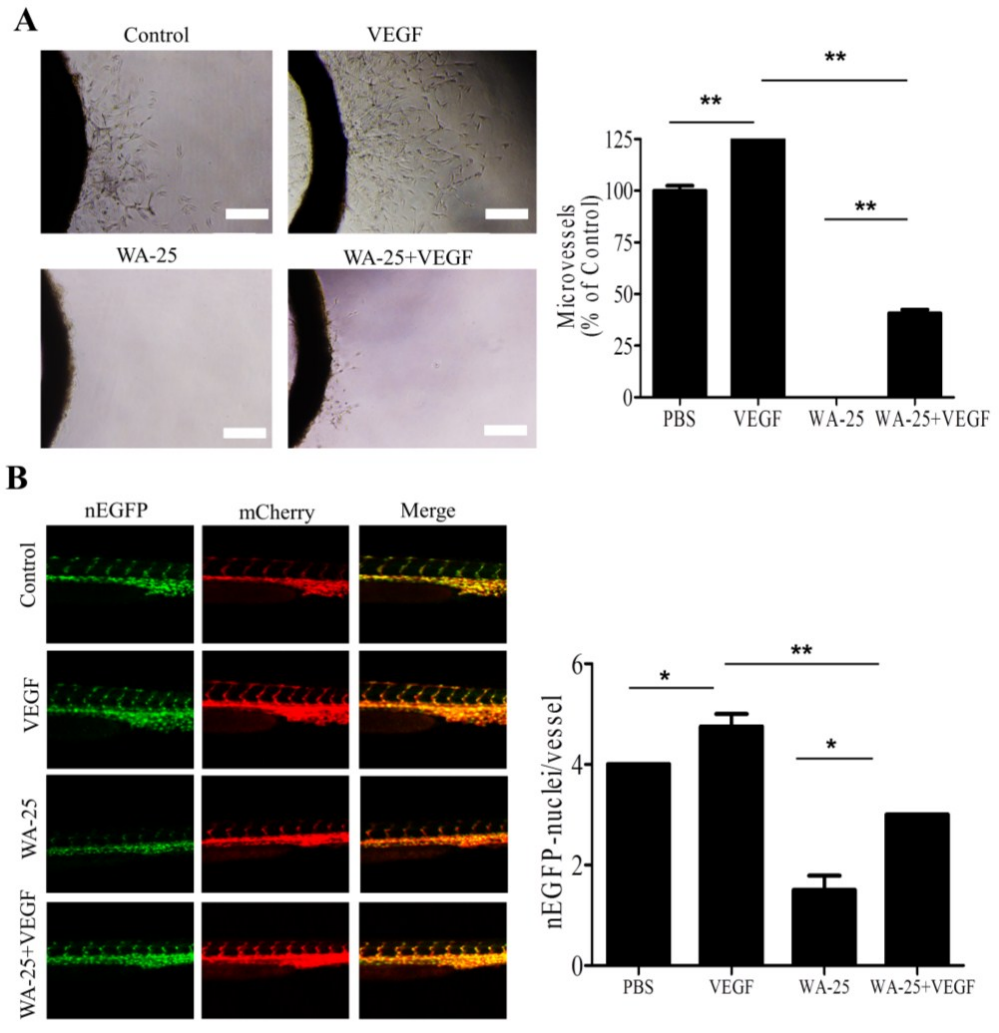
Effect of VEGF-A supply on WA-25-induced neovascularization blockade *in vivo* (**A**) Effect of exogenous VEGF-A on WA-25-induced angiogenesis blockade on the microvessel sprouting in aorta rings. Rat aortic rings were placed in Matrigel and treated with VEGF-A or WA-25. The effect of WA-25 on the formation of vessel sprouts from various aortic samples was observed on Day 7; (**B**) Effect of exogenous VEGF-A on WA-25-induced angiogenesis in *Tg(kdrl:mCherry^ci5^-fli1a:negfp)^y7^* zebrafish embryos. Embryos were treated with WA-25 (50 μM) at 6 hpf with or without VEGF (10 ng/mL) and then imaged at 24 hpf. The number of endothelial cells on the ISV was determined by counting the green nuclei over the red blood vessel. Data are represented as mean ± SD (*n* = 12). Asterisks indicate statistical significance *versus* control (* *p* *<* 0.05 and ** *p* *<* 0.01).

Because exogenous VEGF-A partially alleviated the WA-25-induced VEGF-A downregulation *in vitro*, we evaluated the influence of VEGF-A supply on WA-25-mediated antiangiogenic function *in vivo*. In rat aortic ring assay, VEGF-A supply elicited prominent microvessel sprouting (Figure 6A). In addition, exogenous VEGF-A partially alleviated the WA-25-induced inhibition of vessel sprouting. Consistent with this, exogenous VEGF-A supply partially restored the WA-25-induced reduction of endothelial cells in ISVs of the transgenic zebrafish Tg (kdrl:mCherry^ci5^-fli1a:negfp^y7^) (Figure 6B).

## 3. Discussion

The present study reveals the novel anti-angiogenic function and mechanism of WA-25. Because angiogenesis is essential for neointima formation during the pathogenesis of atherosclerosis, the discovery of WA-25 as an angiogenesis inhibitor elucidates how WA-25 administration confers cardiovascular protection in animals with atherosclerosis [23]. In addition to suppressing COX2 and iNOS expression in macrophages, WA-25 attenuates MMP-2/-9 release and VEGF/VEGFR2 expression in endothelial cells to block neovascularization. However, it remains to be determined whether WA-25 exerts an influence on other cell type in the vascular system, such as smooth muscle cells or cardiomyocytes, to alleviate heart diseases. The nuclear factor kappa B (NFκB) pathway regulates the expression of iNOS, COX2, MMP and VEGF/VEGFR2 [26]. Additional studies may be required to delineate whether WA-25 modulates the expression of these pro-inflammatory and pro-angiogenic genes through the NFκB pathway.

MMP constitute a large family of zinc-binding endopeptidases that play a pivotal role in extracellular matrix degradation, invasion, metastasis, and angiogenesis. Particularly, MMP-2 and MMP-9 are the key gelatinases that regulate angiogenic responses in endothelial cells [27,28]. In the present study, WA-25 treatment preferentially inhibited MMP-9 release in endothelial cells whereas it exerted a lesser influence on MMP-2 expression. This seems in to be consistent with our recent study on lung cancer, in which WA-25 potently inhibited MMP-9 expression in human A549 and murine Lewis lung carcinoma cells, thereby suppressing lung cancer growth in animal models [29]. The indication of WA-25 as an MMP inhibitor deserves a detailed investigation for future clinical development.

Angiogenesis can be divided into the following steps: Endothelial proliferation, migration, and interaction with extracellular matrix/mural cells. This study provides critical insights into how WA-25 regulates angiogenesis at distinct angiogenic steps. Unlike many anti-angiogenic agents, the toxicity of WA-25 to endothelial cells was relatively low. Moreover, WA-25 application was highly tolerated in zebrafish embryos throughout the 7-day experimental period. Despite having low cytotoxicity, WA-25 is a potent inhibitor of other angiogenic processes, particularly migration and tube formation, with an IC_50_ in the range of 5–10 μM. Thus, the features of high anti-angiogenic potency and low toxicity advocate the therapeutic potential of WA-25 for the treatment of angiogenesis-dependent diseases.

Recently, VEGF/VEGFR2 signaling has been one of the most explored pathways for drug development (for examples, bevacizumab and ranibizumab) [26,30]. One crucial finding of this study is that the inhibition of the VEGF/VEGFR2 signaling pathway contributes to the anti-angiogenic mechanism of WA-25. Thus, WA-25 may also act as a novel antagonist of VEGF/VEGFR2 signaling. However, because the VEGF supply only partially reversed the antiangiogenic effects of WA-25, an additional mechanism may be involved in the anti-angiogenic function of WA-25. One likely candidate is nitric oxide (NO) signaling, which is also critical to angiogenesis. Pilot studies conducted by our group revealed that WA-25 treatment influenced NO homeostasis and modulated endothelial nitric oxide synthetase expression in endothelial cells [31]. Future studies are warranted to elucidate the anti-angiogenic mechanism of WA-25.

## 4. Methods and Materials

### 4.1. Coral Compounds and Antibodies

WA-25 (dihydroaustrasulfone alcohol) was synthesized by the Research Center of National Research Program for Biopharmaceuticals (Taipei, Taiwan) as described previously [23]. Quercetin and VEGF-A was obtained from Sigma Chemical (St. Louis, MO, USA). All drugs were dissolved in normal saline. Antibodies against VEGF (SC-152), VEGFR2 (SC-6251), and β-actin (SC-8432) were obtained from Santa Cruz Biotechnology, Inc. (Santa Cruz, CA, USA).

### 4.2. Aortic Ring Assay

The *ex vivo* angiogenesis assay was performed as described previously [32]. Thoracic aortas were removed from Sprague-Dawley rats (male; 8-week-old) and immediately transferred to a culture dish containing ice-cold serum-free MCDB131 media (Life Technologies Ltd., Paisley, Scotland). The periaortic fibroadipose tissue was carefully removed with microdissecting forceps so as not to damage the aortic wall. Each aortic ring was sectioned and washed five times in MCDB131 media. Ring-shaped explants of aorta were then embedded in the 1 mL mixtures of Matrigel and MCDB131 (1:1). Then, the aortic rings were polymerized and kept in triplicate at 37 °C in 24-well culture plates. After polymerization, each well was added with 1 mL of MCDB131 (Life Technologies Ltd., Paisley, Scotland) supplemented with 25 mM NaHCO_3_, 2.5% rat serum, 1% glutamine, 100 U/mL penicillin, 100 μg/mL streptomycin and WA-25 (1–20 μM) to the upper on Matrigel-based embedded aortic ring in each well. The rings were incubated at 37 °C in a humidified environment for 7 days and the vascular sprouting was examined using a microscope equipped with a digital imaging system (Olympus; Tokyo, Japan). The greatest distance from the aortic ring body to the end of the vascular sprouts (sprout length) was measured using the NIH Image program at three distinct points per ring. All animal experiments were approved by the National Sun Yat-sen University Animal Care and Use Committee and complied with the Guiding Principles in the Care and Use of Animals of the American Physiology Society.

### 4.3. Zebrafish Angiogenesis Model

Zebrafish (*Danio rerio*) transgenic lines: Tg(fli-1:EGFP)^y1^, in which the enhanced green fluorescent proteins (EGFP) is expressed in all endothelial cells of the vasculature [33,34] and Tg(kdrl:mCherry^ci5^-fli1a:negfp^y7^), in which endothelial cells are labeled with green nuclei by nuclear enhanced green fluorescent protein (nEGFP) expression and the vessels are labeled with red by cytoplasmic mCherry expression, were obtained from the Taiwan Zebrafish Core Facility (Academia Sinica, Taipei, Taiwan). Zebrafish were raised and maintained at the 28.5 °C incubator accordance with The Zebrafish Book [35] with approval from the National Sun Yat-Sen University Animal Care Committee. Embryos were treated with 0.003% 1-phenyl-2-thiourea (PTU; Sigma, Schnelldorf, Germany) at 6 h post fertilization (hpf) to prevent pigment formation [36,37,38] and used to monitor the effects of WA-25 on embryonic angiogenesis. Zebrafish embryos were generated through natural pair-wise mating and raised at 28 °C in embryo water (0.2 g/L Instant Ocean Salt in distilled water). Approximately 20 healthy embryos were placed in 6cm dishes and various concentrations of WA-25 were separately added into the embryo water at 6 hpf. The embryo water containing WA-25 was replaced daily. At 72 hpf, the embryos were anesthetized using 0.05% 2-phenoxyethanol in embryo water. The embryos were further observed for blood vessel development, particularly in the intersegmental vessels (ISVs) and subintestinal vessel plexus (SIV), by using a microscope with a digital imaging system.

### 4.4. Cell Culture

Human umbilical vein endothelial cells (HUVECs) were isolated from umbilical veins and cultured in M199 medium (Life Technologies, Gaithersburg, MD, USA) as described previously [38].

### 4.5. Gelatin Zymography

The matrix metalloproteinases (MMPs) secretion in endothelial cells was measured using gelatin zymography [39]. In brief, HUVECs at approximately 80% confluence were supplemented with serum-free media and treated with WA-25 for 24 h. Aliquots of the conditioned media were subjected to separation with 10% SDS-PAGE, the gel containing 0.1% type-A gelatin (Sigma, Schnelldorf, Germany). After electrophoresis, the gel was washed twice with 2.5% Triton X-100, incubated in a buffer containing 40 mM Tris-HCl, pH 8.0; 10 mM CaCl_2_; and 0.01% sodium azide at 37 °C for 18–24 h, stained with 0.25% Coomassie blue R-250 in 50% methanol and 10% acetic acid for 1 h, and destained with 10% acetic acid and 20% methanol. The gelatinolytic regions created by MMPs were visualized as white bands with a blue background and quantified using a densitometer.

### 4.6. Proliferation Assay

Cell viability was measured using a quantitative colorimetric assay, the 3-[4,5-dimethylthiazol-2-yl]-2,5-diphenyl-tetrazolium bromide (MTT) assay [39]. HUVECs were cultured in a 24-well plate at a density of 4 × 10^4^ cells/mL overnight. Cells were incubated in M199 medium containing 0.5 mg/mL of MTT for 2 h at 37 °C. The formazan in viable cells was dissolved in dimethylsulfoxide and determined by reading optical densities on a microplate reader (DYNEX Technologies Inc., Chantilly, VA, USA) at an absorption wavelength of 570 nm.

### 4.7. Migration Assay

The cell migration assay was performed as described previously [40]. HUVECs were seeded in triplicate in the upper compartment of the chamber (2.5 × 10^4^ cells/50 μL per well) and supplemented with serum free M199 media. The lower compartment was filled with 30 μL of M199 media containing 10% fetal bovine serum (FBS) serum media. A polycarbonate filter (8-μm pore size Nucleopore; Costar, Cambridge, MA, USA) coated with 0.1% gelatin to allow cell adhesion was used to separate the compartments. After incubation for 4 h in a humidified 5% CO_2_ atmosphere chamber at 37 °C, cells on the upper side of the filter migrated to lower side. Migrated cells were fixed in absolute methanol and stained with 10% Giemsa solution (Sigma, Schnelldorf, Germany). Finally, the fixed cells were photographed using a microscope with a digital imaging system, and counted as mean ± standard deviation (SD) per filter under five high-power fields.

### 4.8. Tube Formation Assay

The tube formation assay was performed as described previously [40]. Matrigel (Becton Dickinson, Bedford, MA, USA) was diluted with cold serum-free M199 media to 10 mg/mL. The diluted Matrigel solution was added to 96-well plates (70 μL per well) and allowed to form a gel at 37 °C for 1 h. Cell suspensions (3 × 10^4^ cells/70 μL per well) in M199 media containing 10% FBS were plated on Matrigel-coated wells and incubated for 6–8 h at 37 °C in 5% CO_2_. After incubation, the endothelial tubes were observed and photographed using a microscope with a digital images system.

### 4.9. Immunofluorescence Assay

To observe the expression of WA-25, VEGF and VEGFR2 on WA-25-treated endothelial cells, immunofluorescence staining was performed as described previously [41]. After treatment with WA-25 (20 μM) for 48 h, the fixed HUVECs were permeabilized using buffer containing 0.1% normal goat serum and 0.1% Triton X-100 in PBS, and incubated with the VEGF or VEGFR2 antibody (1:100 dilution) at 4 °C overnight. The cells were then washed three times with PBS and incubated with the corresponding Alexa-488-conjugated (or Alexa-546-conjugated) secondary antibody (1:1000 dilution; Molecular Probes) for 1 h at room temperature. Finally, the cells were rinsed twice with PBS and incubated with DAPI for 5 min. After mounting in anti-Fade media (Invitrogen, Carlsbad, CA, USA), the fluorescence images of cells were captured using a ZEISS LSM PASCAL multiphoton confocal microscope image system (Carl Zeiss, Jena, Germany).

### 4.10. Flow Cytometric Analysis

The surface VEGFR2 expression in HUVECs was determined using the flow cytometric analysis [34]. After treatment with PBS or WA-25 (20 μM) for 48 h, HUVECs were trypsinized and incubated with the VEGFR2 antibody (1:200 dilution) in PBS at 4 °C for 2 h. After being washed twice with PBS, cells were incubated with the Alexa-488-conjugated secondary antibody (1:100 dilution; Molecular Probes) at 4 °C for 1 h. Finally, the cells were washed twice with PBS, and resuspended in PBS for analysis in a flow cytometer (BD Biosciences; San Jose, CA, USA).

### 4.11. Quantitative Reverse Transcription-Polymerase Chain Reaction (qRT-PCR)

HUVECs were homogenized with theTRIzol reagent (TEL-TEST, Inc., Friendswoods, TX, USA) to extract total RNA. Subsequently, 5 μg of the total RNA was used for the reverse transcription with Superscriptase III (Invitrogen; Carlsbad, CA, USA) using oligo-dT and random primers. The cDNA was then used for real-time PCR that performed in a Lightcycler (Roche, Mannheim, Germany) using a SYBR green assay. The PCR reaction was performed in SYBR Green PCR Master Mix (Roche, Mannheim, Germany) following protocols provided by the manufacturer. The primer sequences for VEGF were forward5′-CCCTGATGAGATCGAGTACA-3′ and reverse 5′-AGGAAGCTCATCTCTCCTAT-3′.The primer sequences for VEGFR2were forward 5′-TCATTATTCTAGTAGGCACGGCG-3′ and reverse 5′-GACAAGTAGCCTGTCTTCAGTT-3′.The primer sequences for MMP-2 were forward 5′-TCTCCTGACATTGACCTTGGC-3′ and reverse 5′-CAAGGTGCTGGCTGAGTAGATC-3′. The primer sequences for MMP-9 were forward 5′-CTTTGACAGCGACAAGAAGTGG-3′ and reverse 5′-GGCACTGAGGAATGATCTAAGC-3′. The expression was normalized to that of β-actin: With the following primer sequences: Forward 5′-TCACCCACACTGTGCCCATCTACGA-3′, primer 5′-CAGCGGAACCGCTC ATTGCCAATGG-3′.

### 4.12. Western Blot Analysis

HUVEC lysates were prepared using the RIPA lysis buffer (50 mM Tris-HCl pH 7.4, 1% NP-40, 0.25% sodium deoxycholate, 150 mM NaCl, 1 mM PMSF and protease inhibitors). An aliquot of proteins was separated using 10% sodium dodecyl sulfate-polyacrylamide gel (SDS-PAGE) and transferred onto the polyvinylidene difluoride membranes (PVDF) (Immobilon-P membrane; Millipore, Bedford, MA, USA). After blocking for 30 min, the membrane was incubated with primary antibodies for 2 h at room temperature and then with horseradish peroxidase (HRP)-conjugated secondary antibodies (Vector Laboratoriess, Burlingame CA, USA) (1:5000 dilution) for 1 h. Immunoreactivity was detected using ECL plus luminol solution (Amersham Biosciences, Piscataway, NJ, USA). The immunoband intensities were quantified by densitometric scanning. The primary antibodies used in this study were those antibodies against VEGF, and VEGFR2, (1:1000 dilution) as well as β-actin (1:5000 dilution).

### 4.13. Enzyme-Linked Immunosorbent Assay (ELISA)

The VEGF release from HUVECs was measured using VEGF ELISA kit (R&D Systems Inc. Minneapolis, MN, USA). After WA-25 (1–20 μM) treatment for 24 h, VEGF concentrations in the cultured media of HUVECs were measured using the VEGF ELISA kit by following protocols provided by the manufacturer.

### 4.14. Statistical Analysis

All values are expressed as means ± SD A paired t test was used to statistically assess the differences between the groups. The differences were considered to be statistically significant when *p* < 0.05.

## 5. Conclusions

WA-25 potently inhibits angiogenesis *in vitro* and *in vivo* by eliciting the blockade of MMP-2/MMP-9 expression and VEGF/VEGFR2 signaling pathway in endothelial cells. Given the pivotal role of VEGF/VEGFR2 signaling in pathological angiogenesis, WA-25 may serve as a novel therapeutic agent for diseases caused by excessive angiogenesis.
